# Pancreatoscopy-Guided Retrieval of a Migrated Stent Using a Novel Thin Pancreatoscope in a Patient with Roux-en-Y Gastrectomy

**DOI:** 10.1055/a-2883-0681

**Published:** 2026-06-12

**Authors:** Ryuichi Watanabe, Yuki Tanisaka, Shomei Ryozawa, Masafumi Mizuide, Akashi Fujita, Suguru Ito, Ryosuke Hamamura

**Affiliations:** 1 Gastroenterology 183786 Saitama Medical University International Medical CenterHidakaSaitamaJapan

## A case description



**Video 1** Successful pancreatoscopy-guided retrieval of a migrated stent using a novel thin cholangioscope under a balloon enteroscope in a patient with Roux-en-Y gastrectomy.


**Fig. 1:**
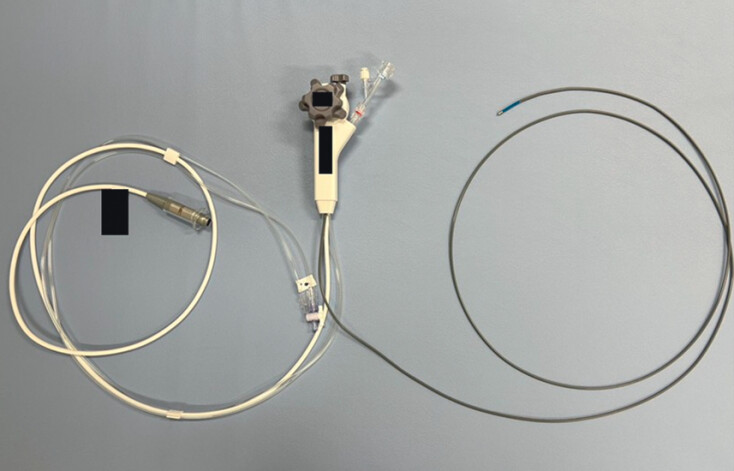
A thin cholangiopancreatoscope (eyeMAX; Micro-Tech, China) measuring 219 cm in length and 9-Fr in diameter.

Pancreatic duct (PD) stent placement can be performed to prevent post-endoscopic retrograde cholangiopancreatography (ERCP) pancreatitis (PEP) 1​ . However, stent migration may also occur after PD stent placement. Although the endoscopic retrieval of migrated PD stents has been widely reported 2​ ​3​ , retrieval in patients with Roux-en-Y gastrectomy under balloon enteroscopy is considered particularly challenging. Recently, a novel thin cholangiopancreatoscope (eyeMAX; Micro-Tech, China), with a length of 219 cm and a diameter of 9-Fr ( Fig. 1 ), has enabled cholangiopancreatoscopy-guided interventions to be performed using a balloon enteroscope 4​ . We report a case of successful pancreatoscopy-guided retrieval of a migrated stent using a novel thin pancreatoscope under balloon enteroscopy in a patient with Roux-en-Y gastrectomy.

**Fig. 2:**
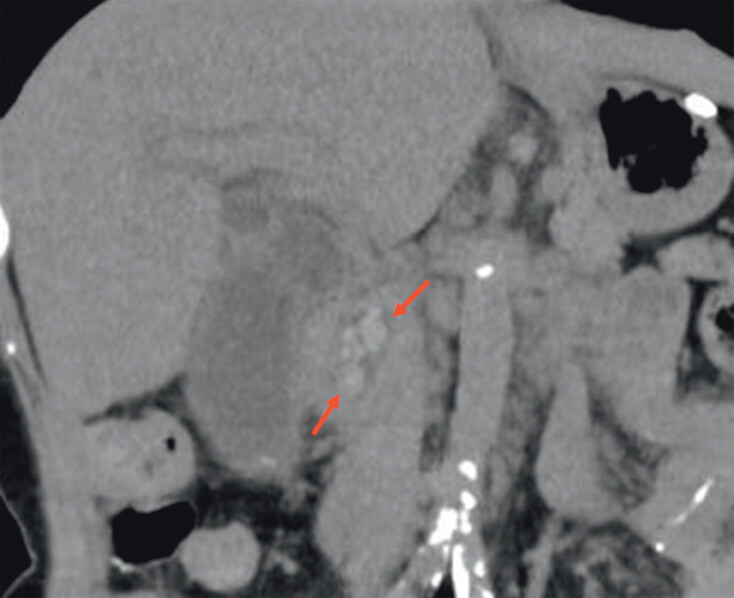
Computed tomography revealing multiple stones in the common bile duct (red arrow).

**Fig 3:**
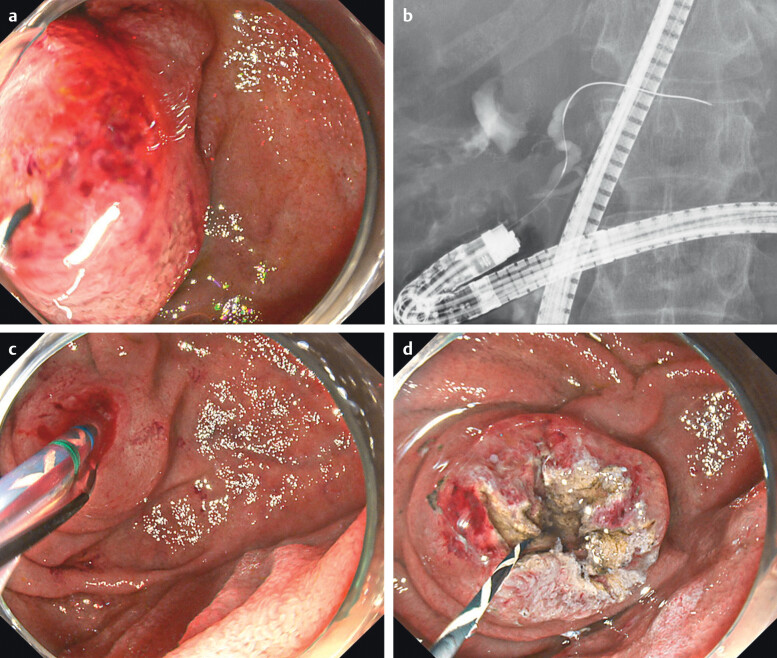
Endoscopic and fluoroscopic findings. (a and b) Only pancreatic duct cannulation was achieved. (c and d) Transpancreatic biliary sphincterotomy was performed; however, selective biliary cannulation was not achieved.

**Fig. 4:**
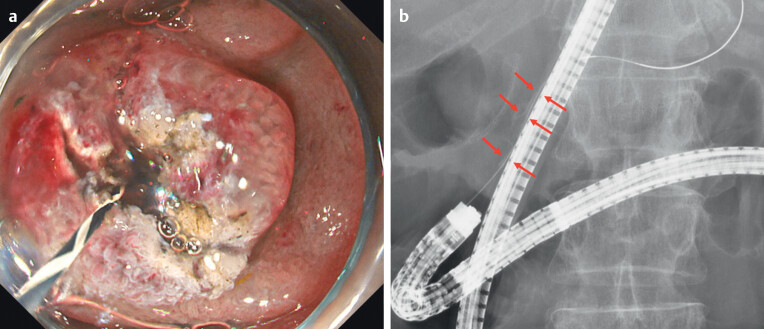
Endoscopic and fluoroscopic findings revealing the plastic stent migrated into the bile duct (red arrow).

**Fig. 5:**
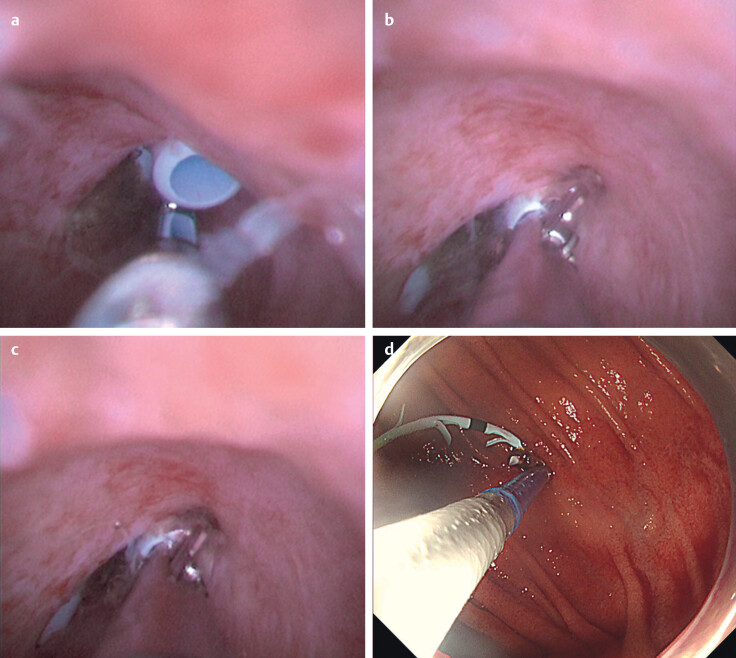
Pancreatoscopic and endoscopic findings for stent retrieval. ( **a** ). Pancreatoscopy revealing the migrated stent in the pancreatic duct. ( **b** and **c** ) The distal end of the stent was successfully grasped under direct pancreatoscopic visualization. ( **d** ). Endoscopic finding revealing the successful stent retrieval of a migrated stent.

A 62-year-old woman with common bile duct stones, who had previously undergone Roux-en-Y gastrectomy, was referred to our institution ( Fig. 2 ). ERCP was performed using a short-type single-balloon enteroscope (SIF-H290; Olympus Marketing, Japan) with a working length of 152 cm and a 3.2-mm working channel 5​ . As only pancreatic duct cannulation was achieved, the double-guidewire technique and transpancreatic biliary sphincterotomy were performed; however, selective biliary cannulation was not achieved ( Fig. 3 ). Therefore, the 5-Fr PD stent was placed to prevent PEP. However, stent migration occurred during the procedure ( Fig. 4 ). Because the fluoroscopy-guided stent retrieval was considered challenging under balloon enteroscopy, pancreatoscopy-guided stent retrieval was attempted using a novel thin pancreatoscope ( Video 1 ). The migrated stent was clearly visualized under pancreatoscopy ( Fig. 5a ). Therefore, we attempted to retrieve the stent using biopsy forceps that could be passed through the thin pancreatoscope. The distal end of the stent was successfully grasped under direct pancreatoscopic visualization ( Fig. 5b, c ), and the stent was successfully retrieved ( Fig. 5d ).

This case highlights that pancreatoscopy-guided retrieval using a novel thin pancreatoscope under balloon enteroscopy may be an effective option for managing migrated PD stents in such cases.

Endoscopy_UCTN_Code_TTT_1AR_2AK

## Conflict of interest

I declare that the authors have not had any economic or personal ties as outlined in “Information on possible conflicts of interest” during the past 3 years.
